# 

**Published:** 1918-04

**Authors:** 


### ANNOUNCEMENTS

The next regular meeting of the Michigan State Board of Dental Examiners will be held in the city of Ann Arbor, in the Dental Building of the Dental Department of the University of Michigan, June 18 to 23, both inclusive. This meeting will be held for the purpose of offering an examination to all eligible candidates who wish to apply for a license to practice dentistry in the state of Michigan. All candidates must furnish the secretary with application and fee not later than ten days prior to examination. Applications and all other information may be obtained by addressing the Secretary-Treasurer, E. O. Gillespie, Stephenson, Mich.

The next meeting of the Iowa State Board for the examination of applicants will be held at Iowa City, June 3, commencing at 9:00 a. m.

For further information address the secretary, Dr. J. A. West, 417 Utica Bldg., Des Moines, Iowa.

*National Association of Dental Faculties.* The next annual meeting of the National Association of Dental Faculties will be held in the green room of the Congress Hotel, Chicago, Ill., August second, at noon. The Executive Committee will meet at 10:00 a. m. on the second. The meeting will continue through August third. Charles Channing Allen, Sec'y., N. W. Cor. 10th and Troost, Kansas City, Mo., care K. C. Dental College.

*New York State Society.* Will celebrate its fiftieth anniversary in Saratoga, June 13-15. A great meeting is arranged and the profession is cordially invited. A. P. Burkhart, Sec'y., Auburn, N. Y.

*Indiana State Meeting* will be held May 21-23 in Indianapolis, Ind.

*Illinois State Meeting* will be held May 14-17 in Bloomington, Ills.

